# Electron Scattering by Tetrahydrofuran Molecules: Elastic and Electronically Inelastic Interactions

**DOI:** 10.1021/acs.jpca.5c06372

**Published:** 2025-11-17

**Authors:** Yan A. C. de Avó, Giseli M. Moreira, Romarly F. da Costa

**Affiliations:** † Centro de Ciências Naturais e Humanas, 74362 Universidade Federal do ABC, 09210-580 Santo André, São Paulo, Brazil; ‡ Departamento de Física, Universidade Estadual do Centro-Oeste, 85040-167 Guarapuava, Paraná, Brazil; § Departamento de Física, Universidade Federal do Paraná, Caixa Postal 19044, 81531-980 Curitiba, Paraná, Brazil

## Abstract

We report elastic and electronically inelastic cross sections for scattering of electrons with energies up to 30 eV by the tetrahydrofuran molecule in the gas phase. The calculations were performed by using the Schwinger multichannel method implemented with norm-conserving pseudopotentials. We analyzed five distinct scattering models to explore the impact of multichannel coupling effects on the description of elastic and electronically inelastic collisions, each incorporating a different channel coupling scheme. Our computed elastic cross sections exhibit strong agreement with existing experimental data. The present results exhibit three resonances of the π*—character and the respective positions are in excellent agreement with the literature. For electronically inelastic processes, we find that the number of coupled channels is the dominant factor in suppressing cross-section magnitudes, with exhaustive channel coupling providing the most rigorous benchmark for these previously uncharacterized individual excitation channels.

## Graphical abstract

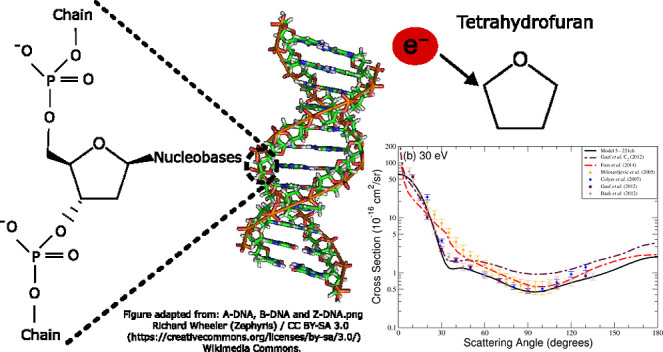

## 1 Introduction

The interaction of low-energy electrons with biomolecular systems plays a crucial role in the understanding of radiation damage mechanisms, particularly those linked to the production of permanent lesions in DNA,^1–3^ with important implications in the context of cancer therapy. Secondary electrons generated by ionizing radiation, most abundantly those with energies in the 1–20 eV range, induce strand breaks and other DNA lesions through direct ionization, electronic excitation, and resonant electron attachment processes.^4,5^ Assessing the biological impact of these interactions at the molecular level requires the determination of accurate cross sections for electron scattering with DNA constituents and their structural analogs. Among these, tetrahydrofuran (THF, C_4_H_8_O) serves as the simplest surrogate for 2-deoxyribose (Figure 1), the sugar moiety present in the DNA backbone.^6,7^ In particular, the DNA backbone can be viewed as a chain of tetrahydrofuran units linked by phosphate groups, with the nucleobases connected to this structure.^6,8^ This makes THF a key system for modeling backbone-centered lesions (e.g., sugar–phosphate cleavage) induced by secondary electrons.

**Figure 1:**
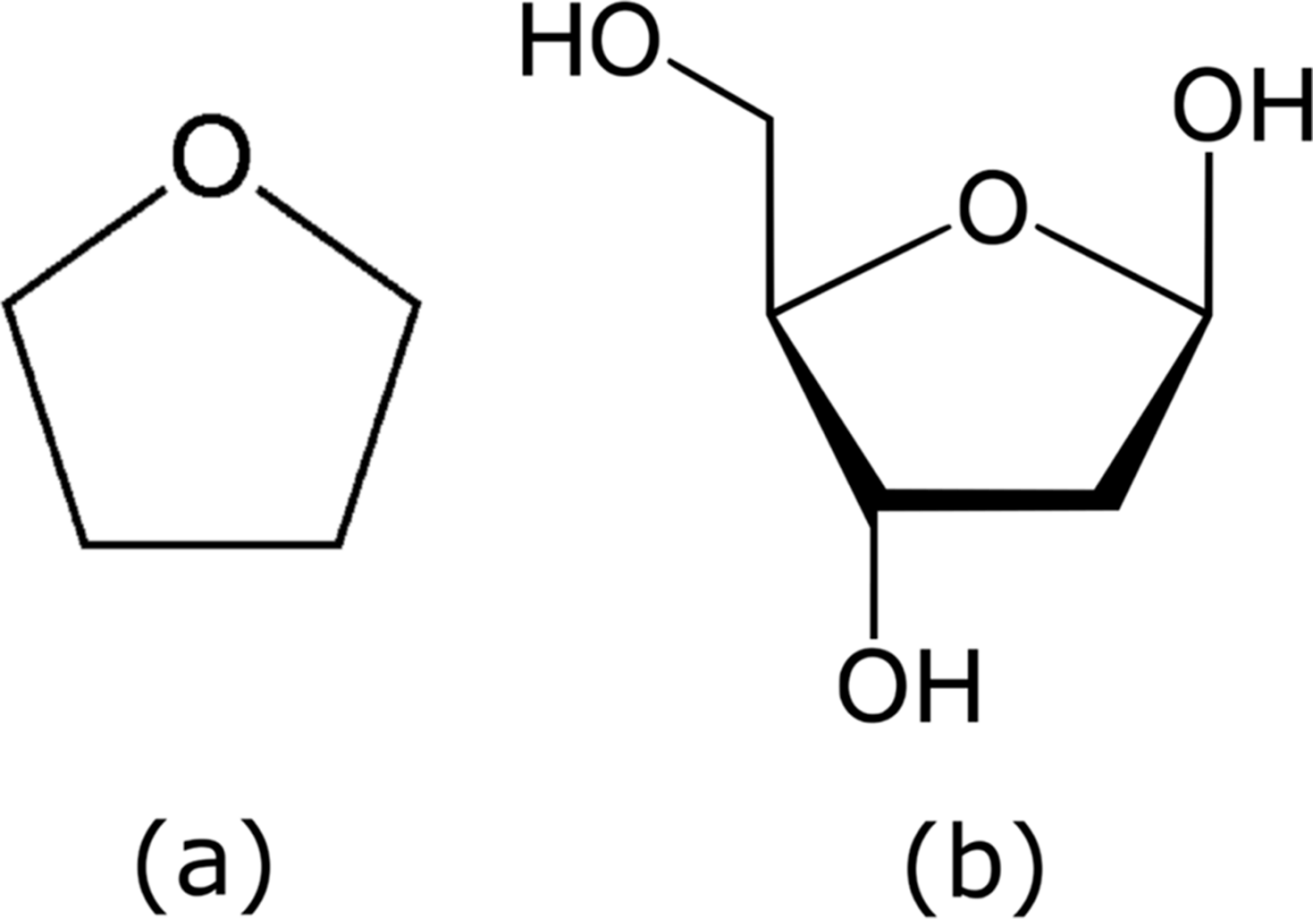
Simplified stick representations of (a) THF and (b) 2-deoxyribose.

Although a comprehensive review of the literature can be found in refs 9,10 , some aspects of the state of the art of electron collisions by the THF molecule that guide the investigations conducted in this work are briefly discussed below.

The equilibrium geometry of THF has been extensively characterized. Calculations show that the *C*_2_ and *C*_s_ conformers are more stable than the *C*_2v_ structure by approximately 0.2 eV, making them the energetically preferred configurations, while maintaining nearly identical dipole moments (1.63 D).^7,11,12^ Early spectroscopic investigations identified electronic excitation bands in THF at 6.38 and 6.90 eV.

Elastic scattering cross sections for THF have been comprehensively measured and calculated across a broad energy range (0 eV–10 keV).^17,18^ While theory and experiment show excellent agreement below 10 eV and above 50 eV, significant discrepancies (around 20% to 30%) persist in the 10–50 eV range.^16^ These inconsistencies underscore the need for improved treatments of polarization and multichannel coupling effects in electron-molecule scattering calculations. In contrast, the cross sections related to electronically inelastic processes in THF remain poorly characterized, despite their significance in radiation damage processes. While vacuum ultraviolet (VUV) spectroscopy^13^ and electron energy loss spectroscopy (EELS)^14^ have mapped the excitation spectrum, quantitative cross sections for individual excited states are notably absent from the literature.

This work addresses these challenges through a systematic study of low-energy electron-THF collisions using the Schwinger multichannel method (SMC) method with norm-conserving pseudopotentials (SMCPP). We employ five distinct channel-coupling models to evaluate polarization and multichannel coupling effects in the elastic cross section and in the electronically inelastic cross section for the two lowest excited states of the molecule.

The structure of this paper is as follows. In Section 2, we outline the theoretical approach and describe the computational methods employed for the scattering calculations. Our results are presented and analyzed in Section 3, and finally, in Section 4, we conclude with a brief summary of our findings.

## 2 Theory and Computational Details

The elastic and electronically inelastic cross sections were obtained in calculations carried out using the SMC method^19,20^ implemented with the norm-conserving pseudopotentials developed by Bachelet, Hamann, and Schlüter (BHS).^21^ While comprehensive descriptions of the method can be found in the literature,^19,22,23^ we focus here on the key aspects pertinent to the current study.

The SMC method is a well-established formalism that provides a variational framework for calculating the scattering amplitudes in electron/positron collisions with molecules. When applied to electron scattering, the method yields the following expression for the scattering amplitude

1 $$f\left({\mathrm{k⃗}}_{f},{\mathrm{k⃗}}_{i}\right)=-\frac{1}{2\pi }\sum_{m,n}\left\langle S_{{\mathrm{k⃗}}_{f}}\left|V\right|\chi_{m}\right\rangle {\left(d^{-1}\right)}_{mn}\left\langle \chi_{n}\left|V\right|S_{{\mathrm{k⃗}}_{i}}\right\rangle$$

where *V* describes the interaction potential between the incident electron and the target; $|S_{{\mathrm{k⃗}}_{i,f}}\rangle$ represents a solution of the unperturbed Hamiltonian *H*_0_, expressed as the product of a plane wave with momentum ${\mathrm{k⃗}}_{i,f}$ and the target state, where the subscripts *i* and *f* stand for initial and final states, respectively; the trial vectors |χ_*m*_⟩ are constructed as (*N* + 1)-electron configuration state functions (CSFs), built from spin-adapted, antisymmetrized products of target electronic states and projectile scattering orbitals; and *d*_*mn*_ are the matrix elements of the *A* ^(+)^ operator in the {χ_*m*_} basis, given by

2 $$d_{mn}=\left\langle \chi_{m}\left|A^{\left(+\right)}\right|\chi_{n}\right\rangle$$

with the operator *A* ^(+)^ written as

3 $$A^{\left(+\right)}=\frac{1}{2}\left(PV+VP\right)-VG_{P}^{\left(+\right)}V+\frac{1}{N+1}\left[Ĥ-\frac{N+1}{2}\left(ĤP+PĤ\right)\right]$$

In eq 3, *P* is the projection operator onto the open-channel space; *G*_*P*_^(+)^ is the free-particle Green function projected onto the *P*-space; and Ĥ = *E* – *H* represents the collision energy minus the Hamiltonian of the electron-target system, with *H* = *H*_0_ – *V*.

The target ground state geometry, obtained from the NIST database,^24^ was optimized at the second order Møller–Plesset perturbation theory (MP2) with the augmented correlation-consistent (aug-cc-pVDZ) basis set, employing the package GAMESS.^25^ The optimization calculation was performed in the C_*s*_ point group. In Figure 2, the optimized molecular geometry of the target is displayed, as visualized using the MacMolPlt^26^ interface. The single particle basis set employed in our calculations was generated according to Bettega et al.^22^ for oxygen and carbon atoms (Table 1) and according to Dunning^27^ for hydrogen atoms (Table 2). To improve the description of the excited states of THF, we utilized improved virtual orbitals (IVOs),^28^ generated from a cationic Fock operator with charge +1, for both particle and scattering orbitals.

**Figure 2:**
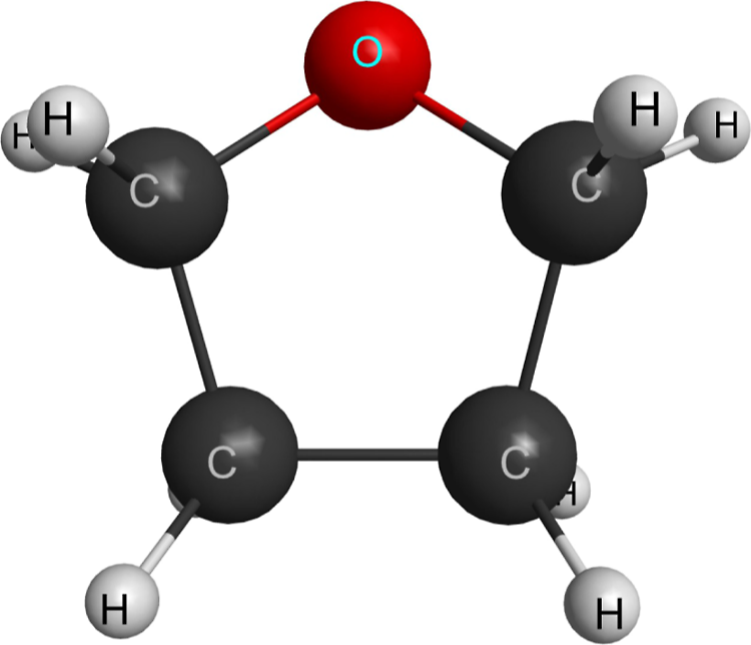
Ball-and-stick model of THF molecule. Generated with MacMolPlt.^26^

**Table 1:** Exponents of the Uncontracted Cartesian Gaussian (CG) Functions Used for Oxygen and Carbon Atoms

type	exponents for oxygen	exponents for carbon
s	16.058780	12.496280
s	5.920242	2.470286
s	1.034907	0.614028
s	0.316843	0.184028
s	0.065203	0.039982
p	10.141270	5.228869
p	2.783023	1.592058
p	0.841010	0.568612
p	0.232940	0.210326
p	0.052211	0.072250
d	0.756793	0.603592
d	0.180759	0.156753

**Table 2:** Exponents and Coefficients of the Cartesian Gaussian (CG) Functions Used for Hydrogen Atoms

type	exponents for hydrogen	coefficients for hydrogen
s	13.361500	0.130844
s	2.013300	0.921539
s	0.453800	1.000000
s	0.123300	1.000000
p	0.750000	1.000000

The scattering calculations were performed according to the minimal orbital basis for single configuration interactions (MOB-SCI) strategy.^29^ This methodology involves representing the target states included in the open-channel space using a minimal basis of spin-adapted Slater determinants, carefully selected to reproduce the energy of the first electronically excited states with high accuracy, compared to our reference calculation, as described in the following. To implement this strategy, the energy spectrum of the electronically excited states was first obtained according to a full single configuration interaction (FSCI) calculation, which accounts for all single excitations of the electrons in the molecule and employs the IVOs to describe the unoccupied molecular orbitals. Subsequently, the excited states included in the scattering calculations were represented using a reduced set of hole-particle pairs that contribute the most to each excited state. In this context, the term “hole” refers to the orbital vacated by an electron in the single excitation to an unoccupied (particle) orbital. Although the number of hole-particle pairs used in the MOB-SCI strategy is smaller than the total number appearing in the FSCI representation, the selected pairs for a given state in the MOB-SCI approximation account for at least 90% of the energy value obtained for that same state in the FSCI calculation. This process ensures consistency between the two approaches used in the description of the excited states of interest. For a given set of *N* hole-particle orbitals, the total number of channels considered is (2*N* + 1), corresponding to the contribution of the ground state, *N* triplet excited states and *N* singlet excited states.

To investigate the role of multichannel coupling effects in the scattering dynamics, we devise five computational models with increasing levels of complexity. These models differed in three key aspects: (i) the number of excited states included in the MOB-SCI approximation; (ii) the sets of hole-particle orbitals used to describe those states; and (iii) the total number of coupled channels used in the scattering calculations and the number of configuration state functions generated for each symmetry. The progression from the simplest to the most complex model involved a significant increase in the number of excited states and hole-particle pairs, with the most comprehensive model incorporating approximately double the number of excited states and orbital pairs compared to the simplest one. The primary goal of this strategy is to evaluate how the progressive inclusion of excited states and channels affects the magnitude and shape of the integral and differential cross sections, the assignment and energy of resonances, and ultimately, the overall agreement of our results with existing theoretical and experimental benchmarks. Below, we provide a detailed description of the computational models employed in this study.

To investigate the influence of the multichannel coupling effects, we constructed five computational models using subsets of the 2895 excited states chosen from the FSCI spectrum of THF. Models 2 and 3 serve as foundational benchmarks, incorporating the 10 lowest energy triplet and the 10 lowest energy singlet excited states per irreducible representation of the C_s_ point group (*A*′ and *A*″), yielding a total of 40 excited states, selected to ensure symmetry balance. We pick up the hole-particle orbital pairs having the highest contributions for each state, yielding a total of 53 pairs for Model 2 and 91 for Model 3. Models 1, 4, and 5 were then constructed using double the number of states (20 per symmetry and spin coupling, 80 total). However, Models 1 and 4 were designed to retain orbital pair counts similar to their simpler counterparts (52 pairs for Model 1 compared to 53 in Model 2; exactly 91 pairs for Models 3 and 4). In contrast, Model 5 was built to maximize the number of channels (221) for states below a 20 eV energy cutoff, which required the use of 110 hole-particle pairs. This progression allows us to evaluate how incremental changes in the density of states and the number of hole-particle pairs influence the scattering dynamics and the resulting cross sections.

A consistent energy cutoff of 20 eV was applied across all models to guarantee that all electronically excited states remain energetically accessible to incident electrons within the range of energies considered in our work. This cutoff also ensures that all relevant scattering channels remain open at or above 20 eV, excluding the very high-energy pseudostates whose inclusion would not significantly affect the cross sections at low energies. A summary of the five multichannel coupling models is provided in Table 3.

**Table 3:** Number of Channels, FSCI Excited States Being Described and Configuration State Functions per Symmetry for Each of the Multichannel Coupling Models Considered in This Work

			CSFs	
calculation	channels	FSCI states	*A*′	*A*″
Model 1	105	80	5109	5120
Model 2	107	40	5156	5266
Model 3	183	40	8779	8977
Model 4	183	80	8834	8922
Model 5	221	80	10695	10728

It is important to note that, before proceeding to the computationally demanding multichannel calculations, we first performed static-exchange (SE) and static-exchange plus polarization (SEP) calculations. These simpler approximations served two important purposes: (i) they provided a valuable test for the quality of our basis set and computational setup; (ii) they established baseline results for comparison with the more sophisticated treatments that incorporate the effects of multichannel coupling.

In the SE approximation we consider that the electronic cloud of the molecule is kept frozen during the collision and, in this case, the CSFs can be written as

4 $$|\chi_{m}\rangle =A_{N+1}|\Phi_{0}\rangle ⊗|\phi_{m}\rangle$$

where $A_{N+1}$ is the antisymmetrizer; |Φ_0_⟩ represents the *N*-electron Slater determinant of the ground state; and |ϕ_*m*_⟩ is a single-particle orbital used to represent the scattering orbitals.

In the SEP approximation, the polarization effects (representing the distortion of the molecular cloud due to the presence of the incident electron) are taken into account in the calculations, and the configuration space is augmented by including CSFs constructed as

5 $$|\chi_{im}\rangle =A_{N+1}|\Phi_{i}\rangle ⊗|\phi_{m}\rangle$$

where |Φ_*i*_⟩ are *N*-electron Slater determinants obtained by carrying out single excitations of the target electrons from the occupied (hole) orbitals to a set of unoccupied (particle) orbitals. These states represent virtual electronic excitations that provide the necessary flexibility in the wave function. In both SE and SEP approximations, the projection operator *P* includes only the ground state of the target

6 $$P=|\Phi_{0}\rangle \langle \Phi_{0}|$$

where |Φ_0_⟩ is the *N*-electron ground state Slater determinant.

In the multichannel coupling approximation, the formalism is extended to include the real excitations, i.e., the electronically excited states of the target that become energetically accessible (open channels) at a given collision energy. The projection operator *P* is expanded to include these states

7 $$P=\sum_{r=0}^{n}|\Phi_{r}\rangle \langle \Phi_{r}|$$

where *n* is the number of open channels. The CSFs are then constructed for each of these open channels

8 $$|\chi_{rm}\rangle =A_{N+1}|\Phi_{r}\rangle ⊗|\phi_{m}\rangle$$

where *r* = 0,...,*n* and |Φ_*r*_⟩ are the *N*-electron electronic eigenstates of the target Hamiltonian, encompassing the ground state (*r* = 0) and its electronically excited states (*r* > 0).

The fundamental distinction between the SEP and multichannel approximations lies in the nature of the states |Φ_*i*_⟩ and |Φ_*r*_⟩. The SEP approximation employs virtual excitations to model polarization correlation, while the multichannel approximation incorporates the real (physical) electronically excited states of the target to describe the coupling between various inelastic scattering channels.

As the number of channels increases across the models, the corresponding number of configurations also grows. This progression allows for a more extensive description of polarization effects, although an excessive number of channels risks introducing overcorrelation. Furthermore, one of the goals of this systematic approach is to determine whether models incorporating a larger number of excited states and hole-particle orbital pairs yield cross sections that converge toward improved agreement with available experimental data, compared to both earlier SMC calculations and our own simpler models.

## 3 Results and Discussion

### 3.1 Elastic Scattering

In Figure 3 we present the differential cross sections (DCSs) obtained with the SMCPP method according to the SE, SEP and multichannel coupling (Models 1–5) approximations at the impact energies of 6 and 30 eV. At 6 eV (Figure 3a), where only the elastic scattering is energetically allowed, the differences between models arise primarily from polarization effects. The multichannel coupling models yield DCS magnitudes intermediate between the SE and SEP results, consistent with their inclusion of varying levels of polarization contributions.

**Figure 3:**
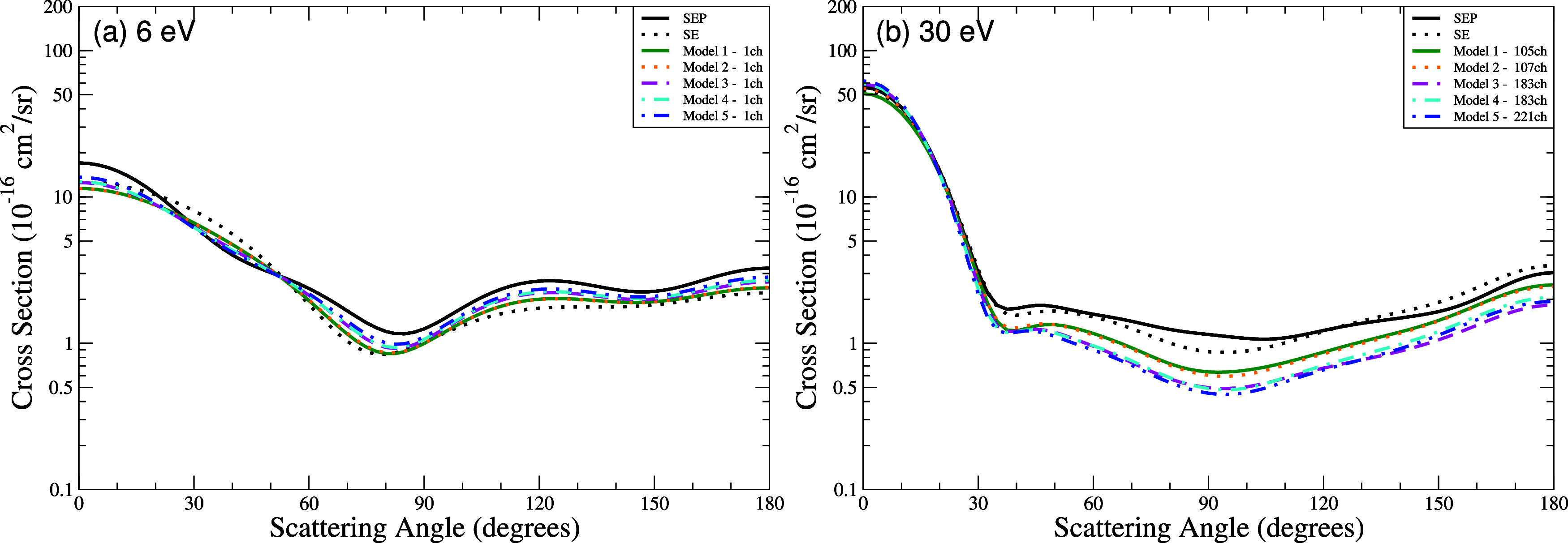
Differential cross section for elastic scattering of electrons by THF at the energies of (a) 6 eV and (b) 30 eV. Calculations were obtained for the SE level (black dashed line) and SEP level (black solid line), as well as for the five models incorporating multichannel coupling effects: 105 channels (solid green line), 107 channels (dotted orange line), 183 channels (dashed magenta line), 183′ channels (dash-dotted cyan line), and 221 channels (dash-dot-dot blue line).

At 30 eV (Figure 3b), the inclusion of multichannel coupling systematically reduces DCS magnitudes across nearly all scattering angles as the number of coupled channels increases. Model 5 (221 channels), which incorporates the largest number of electronically excited states (40) and hole-particle pairs (110), achieves the most significant reduction: its DCS is up to 30% smaller than Model 1 (105 channels, 52 hole-particle pairs) and up to 60% smaller than the SE and SEP approximations. These trends highlight the critical role of channel coupling, which redirects the probability of scattering (flux) into the newly accessible inelastic channels, with Model 5 providing the most comprehensive treatment of open channels within the 20 eV cutoff.

In Figure 4, we present the integral cross sections (ICSs) for elastic scattering over impact energies ranging from 0.1 to 30 eV. Below 1 eV, all models, except SEP, exhibit a pronounced peak, with maxima between 0.2 and 0.5 eV. Notably, Model 5 shows an exceptionally high value at 0.1 eV, over one order of magnitude greater than the SEP result, indicating either an overestimated polarization contribution or a numerical artifact. Above 2 eV, weaker structures appear near 2.8, 3.5, 7.2, 8.5, and 9.4 eV. While all models exhibit these features, their position and intensity vary with the degree of polarization: more polarized models tend to shift the peaks to lower energies, as expected. Above 10 eV, a broad enhancement forms between 10 and 12 eV across all models. In this region, SE and SEP curves display a broader structure compared to the multichannel results.

**Figure 4:**
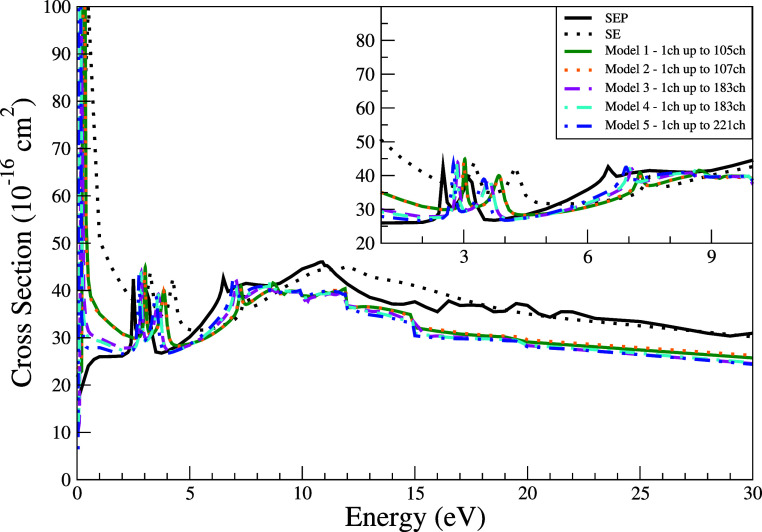
Integral cross section for elastic scattering of electrons by THF at energies ranging from 0.1 to 30 eV. Calculations were obtained for the SE level (black dashed line) and SEP level (black solid line), as well as for the five models incorporating multichannel coupling effects: 105 channels (solid green line), 107 channels (dotted orange line), 183 channels (dashed magenta line), 183′ channels (dash-dotted cyan line), and 221 channels (dash-dot-dot blue line).

Multichannel coupling becomes relevant around 8.6 eV, corresponding to the threshold of the first electronically excited state in our MOB-SCI models (8.3 eV in the FSCI calculation), and becomes dominant beyond 10 eV, when approximately ten inelastic channels are open. We note that our Configuration Interaction Singles (CI-Singles) approach within the SMCPP framework yields higher excitation energies for the lowest-lying excited states of THF compared to more sophisticated models. Specifically, Equation-of-Motion Coupled Cluster Singles and Doubles (EOM-CCSD) calculations^11^ report the first excited state at 6.608 eV, in good agreement with the experimental value of 6.353 eV.^11^ This effect intensifies at higher energies: at 20 and 30 eV, where all inelastic channels are accessible, the discrepancy between multichannel and uncoupled models reaches 20–25%. Among the multichannel models, Model 5 consistently yields the lowest ICS values beyond 12 eV, differing by up to 7% from the others. Despite this deviation, the other models remain within 10% of each other across most of the energy range. These relatively small differences highlight the sensitivity of ICS predictions to the specific treatment of channel coupling and polarization. Nonetheless, models with a comparable number of coupled channels and polarization treatment yield remarkably similar magnitudes and shapes.

Given the overall similarity in ICS and DCS profiles among the multichannel models, and since Model 5 represents our most complete treatment of polarization and multichannel coupling effects, we restrict our detailed comparison with literature data to this model to maintain clarity.

In Figure 5, we present the DCSs for elastic electron scattering by THF at incident energies of 10 eV (Figure 5a) and 30 eV (Figure 5b), calculated using Model 5. For comparison, we include theoretical results from Fuss et al.,^10^ Trevisan et al.,^12^ Gauf et al.^16^ and Winstead and McKoy.^33^ The figure also includes experimental data from Colyer et al.,^8^ Baek et al.,^9^ Allan,^15^ Gauf et al.,^16^ Milosavljević et al.^30^ and Dampc et al.^31^ At 10 eV, Model 5 shows excellent agreement in both shape and magnitude with the theoretical curves of Trevisan et al. and Winstead and McKoy across nearly all angles. Its magnitude is lower than the sharply peaked forward-angle results from Fuss et al. and Gauf et al. Among experimental data sets, the curve corresponding to Model 5 lies closer to the lower end of the reported values, agreeing well with the results obtained by Colyer et al., Gauf et al. and Allan, although it underestimates the prominent forward peak reported by Baek,^9^ which is nearly twice as large below 10°.

**Figure 5:**
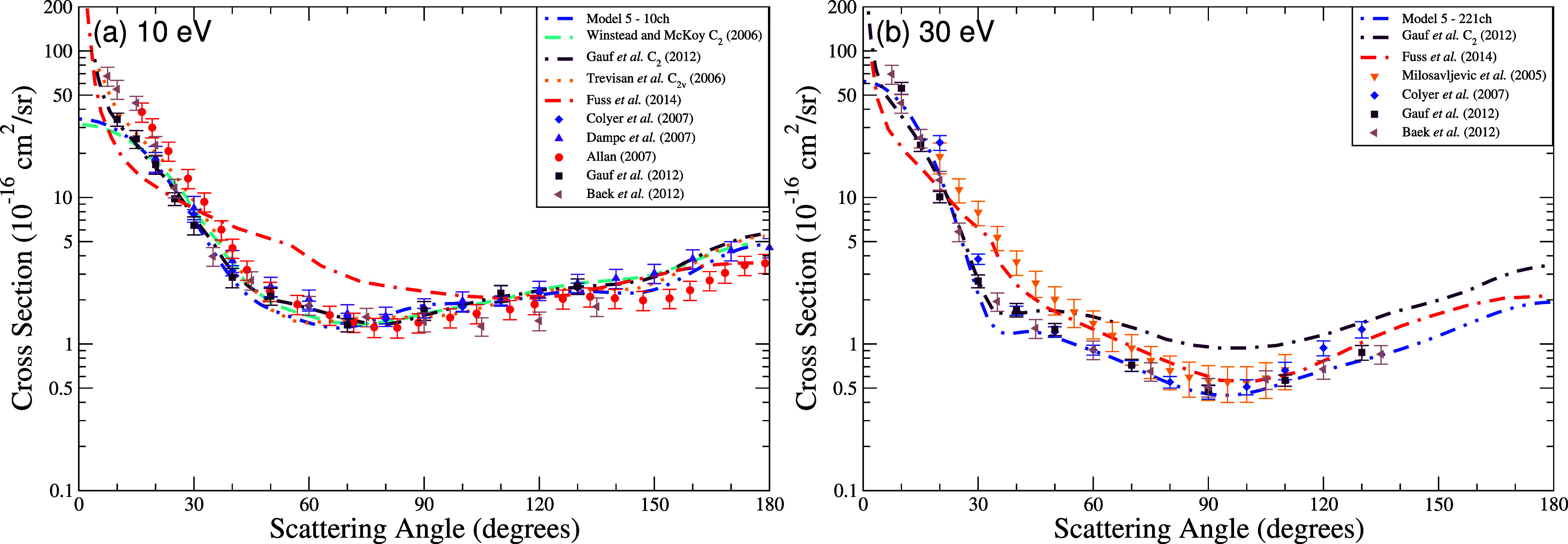
Differential cross section for elastic scattering of electrons by THF at the energies of (a) 10 eV and (b) 30 eV. Calculation was obtained for the model with up to 221 channels (dash-dot-dot blue line). Results from the literature were obtained theoretically by Trevisan et al.^12^ (dotted orange line), Winstead and McKoy^33^ (dashed turquoise line), Gauf et al.^16^ (dash-dotted maroon line) and Fuss et al.^10^ (dash–dash-dot red line) and experimentally by Milosavljević et al.^30^ (orange downward triangle), Colyer et al.^8^ (blue diamond), Dampc et al.^31^ (violet triangle), Allan^15^ (red circle), Gauf et al.^16^ (dark brown square), and Baek et al.^9^ (light brown left-pointing triangle).

At 30 eV, the curve of Model 5 DCS remains in excellent agreement with the theoretical results of Trevisan et al.^12^ and Winstead and McKoy^33^ up to around 80°, and only slightly underestimates the backscattering region beyond that. The DCS associated to Model 5 also aligns very closely with experimental results from Colyer et al.,^8^ Dampc et al.,^31^ and Allan,^15^ capturing both the angular distribution and magnitude particularly well from 30° to 120°. Minor underestimations are visible at larger angles but remain within the experimental error bars.

The integral cross sections for elastic electron scattering by THF, presented in Figure 6 reveal notable differences among both theoretical and experimental data sets. The ICS corresponding to Model 5 aligns closely with the calculations of Fuss et al., differing by less than 5% at energies below 10 eV. Compared to earlier theoretical efforts by Winstead and McKoy^33^ and Gauf et al.,^16^ Model 5 predicts systematically lower magnitudes, with prior models exceeding its values by 50–100% below 10 eV.

**Figure 6:**
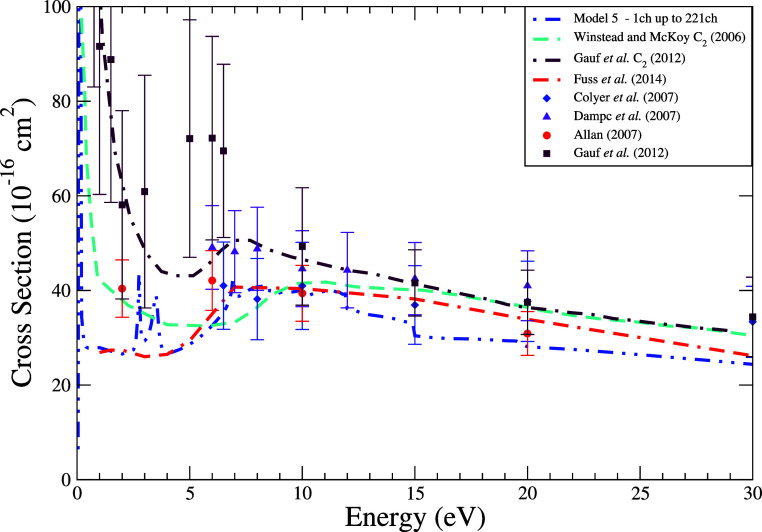
Integral cross section for elastic scattering of electrons by THF at energies ranging from 0.1 to 30 eV. Calculation was obtained for the model with up to 221 channels (dash-dot-dot blue line). Results from the literature were obtained theoretically by Winstead and McKoy^33^ (dashed turquoise line), Gauf et al.^16^ (dash-dotted maroon line) and Fuss et al.^10^ (dash–dash-dot red line) and experimentally by Colyer et al.^8^ (blue diamond), Dampc et al.^31^ (violet triangle), Allan^15^ (red circle) and Gauf et al.^16^ (dark brown square).

These earlier theoretical results, while broadly consistent with experimental trends, often overestimate measured ICS magnitudes. Notably, at 2 eV, Gauf et al. reported a cross section 37% higher than Allan’s experimental value. However, due to the large experimental uncertainties (15–40%, depending on energy and data set), even these discrepancies often fall within reported error bounds. This limits the ability to definitively discriminate between models based solely on ICS magnitude, especially at lower energies. Nonetheless, the Model 5 curve tends to align more closely with the central values of experimental data sets, especially for energies above 5 eV (Figure 7).

**Figure 7:**
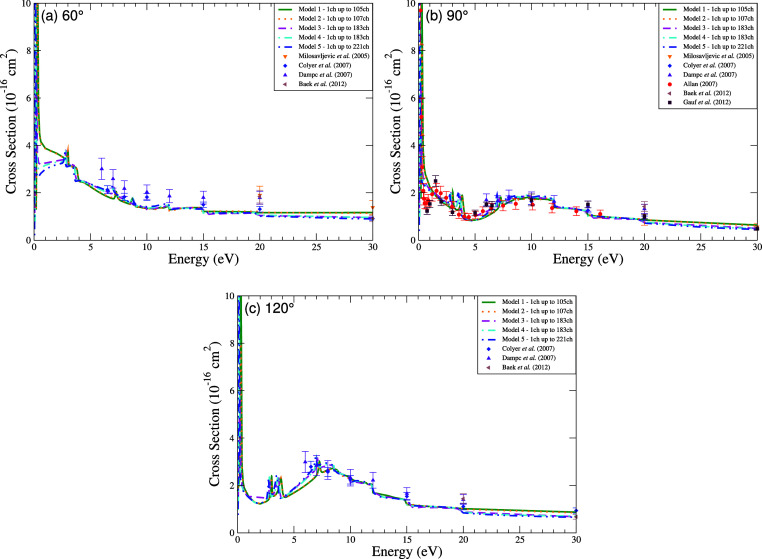
Excitation cross section for elastic scattering of electrons by THF at the scattering angles of (a) 60°, (b) 90°, and (c) 120°. Calculations were obtained for the five models incorporating multichannel coupling effects: 105 channels (solid green line), 107 channels (dotted orange line), 183 channels (dashed magenta line), 183′ channels (dash-dotted cyan line), and 221 channels (dash-dot-dot blue line). Results from the literature were obtained experimentally by Milosavljević et al.^30^ (orange downward triangle), Colyer et al.^8^ (blue diamond), Dampc et al.^31^ (violet triangle), Gauf et al.^16^ (dark brown square), Allan^15^ (red circle), and Baek et al.^9^ (light brown left-pointing triangle).

The low-energy ICS profile (below 15 eV) is marked by resonant structures that we identify as the π_1_^*^, π_2_^*^, and π_3_^*^ temporary anion states. A key finding is the systematic lowering of their energies as the theoretical model incorporates more channels, from Model 1 (105 ch) to Model 5 (221 ch). This behavior is consistent with the expectation that a more comprehensive treatment of polarization and coupling effects leads to more accurate, stabilized resonance positions. The peaks observed in the ranges of 2.75–3.25 eV, 6.94–7.50 eV, and 11.25–12.00 eV are assigned to these resonances based on this systematic behavior and their proximity to known experimental values reported at approximately 2.60 eV,^15^ 6.00–6.50 eV,^8,15,31^ and 10.80 eV,^15^ respectively. Although the identification and characterization of resonant states are not the primary focus of our work, the convergence of our results provides a critical internal benchmark. Ultimately, Model 5 provides the most accurate resonance energies, with its predicted centers (2.75 eV, 6.94 eV, 11.25 eV) showing the best agreement with experiment. This confirms that the exhaustive channel coupling in Model 5 yields the most reliable description of polarization effects for low-energy electron scattering from THF.

### 3.2 Electronically Inelastic Scattering

We now analyze cross sections for electron-impact excitation of the two lowest-energy excited states of THF: the first triplet (^3^ *A*′) and singlet (^1^ *A*′). As theoretical/experimental data for individual excitation channels are unavailable, we compare results across our five multichannel models (Table 3).

The DCS for the first excited state (^3^ *A*′) at 20 eV (Figure 8a) and 30 eV (Figure 8b) exhibit angular distributions with similar shapes, but with magnitudes that vary systematically with the number of coupled channels. Model 5 (221 channels) yields the lowest magnitudes across all angles, with reductions of 40–85% at forward angles (0–30°) and 55–68% at backward angles (180°) compared to Models 1 and 2. At 30 eV, differences diminish but persist: Model 5 remains 8–28% lower at forward angles and 20–50% lower at backward angles. Models 3 and 4 (183 channels) show intermediate reductions (15–30% higher than Model 5).

**Figure 8:**
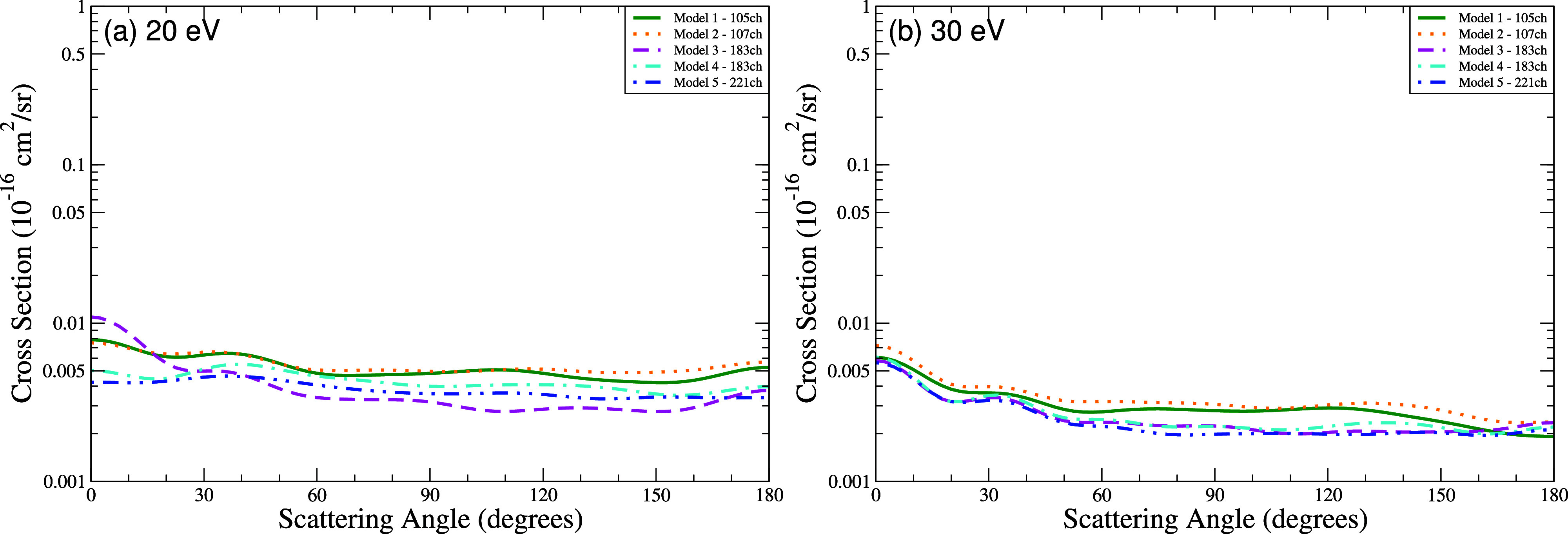
Differential cross section for electronically inelastic scattering of electrons by the THF molecule, specifically for the first electronically excited state of the molecule (^3^ *A*′), at the energies of (a) 20 eV and (b) 30 eV. Calculations were obtained for the five models incorporating multichannel coupling effects: 105 channels (solid green line), 107 channels (dotted orange line), 183 channels (dashed magenta line), 183′ channels (dash-dotted cyan line), and 221 channels (dash-dot-dot blue line).

Notably, models with the same number of excited states but fewer channels (e.g., Models 1 and 4, both with 80 states) differ significantly in magnitude. Models 3 and 4, which share an identical number of channels (183) but differ in the number of excited states (40 and 80, respectively), show only modest differences, demonstrating that the number of coupled channels is a more influential factor in determining the cross section magnitude than the number of excited target states included in the current calculations (Tables 4–6).

**Table 4:** Resonance Positions (in eV) Obtained in the Cross Sections From the SE, SEP and Five Multichannel Coupling Models Considered in This Work^a^

	multichannel coupling models					experiment			theory	
resonances	105 ch	107 ch	183 ch	183′ ch	221 ch	^15^	^31^	^8^	^33^	^16^
π_1_^*^(ICS)	3.03	3.00	2.86	2.83	2.75	2.60	–	–	–	–
π_2_^*^(ICS)	7.28	–	7.08	7.07	6.94	6.20	6.00	6.50	8.00	7.50–8.00
π_3_^*^(ICS)	12.00	–	11.75	11.50	11.25	10.80	–	–	13.00–14.00	12.00–15.00

a The first resonance belongs to the *A*′ symmetry, the second belongs to the *A*″ symmetry and the third belongs to a superposition of both symmetries. For comparison, we include the values measured by Colyer et al.,^8^ Dampc et al.^31^ and Allan,^15^ as well as those calculated by Winstead and McKoy et al.^33^ and Gauf et al.^16^

**5:** DCS Magnitudes and Percentage Differences Relative to Model 5 (221 Channels) at 20 eV^a^

angle (°)	Model 1 (105 ch)	Model 2 (107 ch)	Model 3 (183 ch)	Model 4 (183 ch)	Model 5 (221 ch)
0	0.00787 (+85.6%)	0.00750 (+76.9%)	0.01093 (+157.6%)	0.00501 (+18.2%)	0.00424 (−)
30	0.00613 (+42.6%)	0.00639 (+48.6%)	0.00504 (+17.2%)	0.00449 (+4.4%)	0.00430 (−)
90	0.00481 (+34.4%)	0.00506 (+41.3%)	0.00339 (−5.3%)	0.00399 (+11.5%)	0.00358 (−)
180	0.00526 (+55.6%)	0.00568 (+68.0%)	0.00376 (+11.2%)	0.00397 (+17.5%)	0.00338 (−)

a Values in 10^–16^ cm^2^/sr.

**Table 6:** DCS Magnitudes and Percentage Differences Relative to Model 5 (221 Channels) at 30 eV^a^

angle (°)	Model 1 (105 ch)	Model 2 (107 ch)	Model 3 (183 ch)	Model 4 (183 ch)	Model 5 (221 ch)
0	0.00608 (+8.0%)	0.00722 (+28.2%)	0.00580 (+3.0%)	0.00610 (+8.3%)	0.00563 (−)
30	0.00364 (+14.8%)	0.00394 (+24.3%)	0.00325 (+2.5%)	0.00319 (+0.6%)	0.00317 (−)
90	0.00280 (+39.3%)	0.00300 (+49.3%)	0.00205 (+2.0%)	0.00212 (+5.5%)	0.00201 (−)
180	0.00193 (−9.0%)	0.00237 (+11.8%)	0.00235 (+10.8%)	0.00221 (+4.3%)	0.00212 (−)

a Values in 10^–16^ cm^2^/sr.

The ICS for the ^3^ *A*′ state (Figure 9) displays pronounced structures below 12 eV, with peak magnitudes varying by up to 50% across models. All models show a sharp decline at 12 eV (approximately 50% reduction), followed by a smooth decay to 30 eV. Model 5 exhibits the steepest decline, consistent with its enhanced redistributive scattering from exhaustive channel coupling.

**Figure 9:**
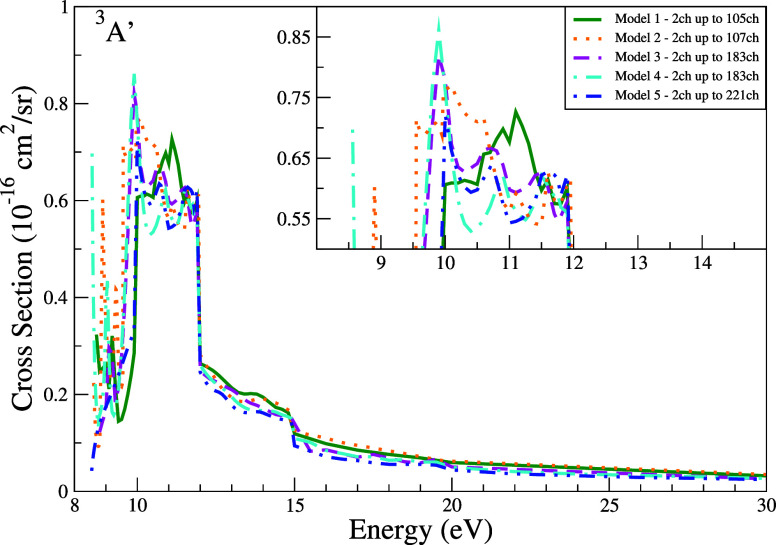
Integral cross section for electronically inelastic scattering of electrons by the THF molecule, specifically for the first electronically excited state of the molecule (^3^ *A*′). Calculations were obtained for the five models incorporating multichannel coupling effects: 105 channels (solid green line), 107 channels (dotted orange line), 183 channels (dashed magenta line), 183′ channels (dash-dotted cyan line), and 221 channels (dash-dot-dot blue line).

For the ^1^ *A*′ state, DCS angular distributions at 20 eV (Figure 10a) and 30 eV (Figure 10b) are more isotropic than those of the ^3^ *A*′ state, with magnitudes 15–25% larger. In a notable contrast to the results for the ^3^ *A*′ state, Model 3 (183 channels, 20 states) yields the lowest cross sections at both energies, around 10–50% below Models 1 and 2. Model 5, while slightly higher than Model 3, remains significantly lower than Models 1 and 2.

**Figure 10:**
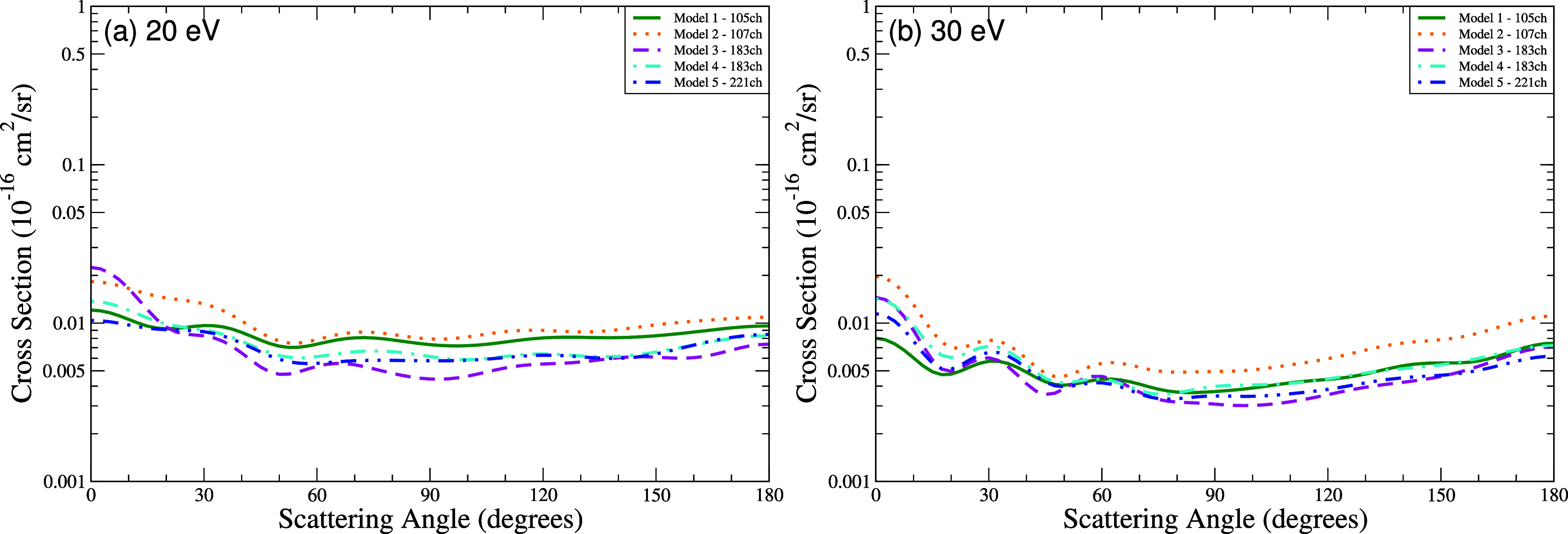
Differential cross section for electronically inelastic scattering of electrons by the THF molecule, specifically for the second electronically excited state of the molecule (^1^ *A*′), at the energies of (a) 20 eV and (b) 30 eV. Calculations were obtained for the five models incorporating multichannel coupling effects: 105 channels (solid green line), 107 channels (dotted orange line), 183 channels (dashed magenta line), 183′ channels (dash-dotted cyan line), and 221 channels (dash-dot-dot blue line).

In particular, models with similar channel counts, such as Models 2 and 3 (20 excited states) and Models 1 and 4 (40 excited states), exhibit more pronounced magnitude differences for the second excited state than were observed for the first one. Despite these state-specific variations, the number of coupled channels remains the primary factor governing the DCS magnitude, a conclusion consistent with our previous findings for the furan molecule^32^

The ICS for the second electronically excited state of THF (^1^ *A*′) is presented in Figure 11. Pronounced structures are observed across all models for energies up to 12 eV, with peak magnitudes varying significantly between them. Out of the five models, Model 5 shows the smoothest profile. At 12 eV, the models undergoes a sharp decline of around 50% of magnitude, followed by a gradual decay up until 30 eV. Similar to the DCS for the second excited state (Figure 10), the ICS magnitude for Model 3 yields slightly lower ICS values than Model 5 at 20 and 30 eV. This contrasts with the first excited state, where Model 5 consistently produced the lowest cross sections.

**Figure 11:**
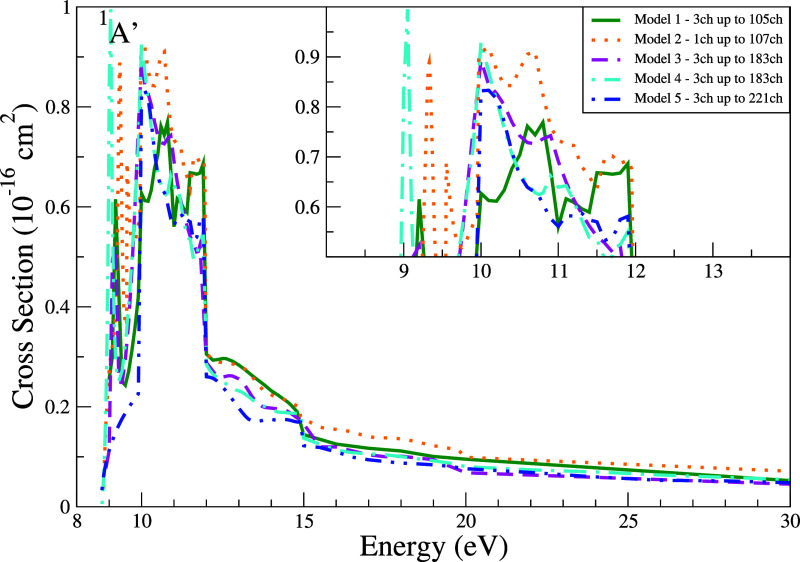
Integral cross section for electronically inelastic scattering of electrons by the THF molecule, specifically for the second electronically excited state of the molecule (^1^ *A*′). Calculations were obtained for the five models incorporating multichannel coupling effects: 105 channels (solid green line), 107 channels (dotted orange line), 183 channels (dashed magenta line), 183′ channels (dash-dotted cyan line), and 221 channels (dash-dot-dot blue line).

## 4 Conclusions

We have presented a comprehensive study on elastic and electronically inelastic electron scattering by THF, a prototype for the sugar moiety in DNA, using the Schwinger multichannel method with norm-conserving pseudopotentials. Five computational models, incorporating 105 to 221 coupled channels, were employed to systematically assess the roles of polarization, multichannel coupling, and excited-state selection in shaping scattering dynamics.

For elastic scattering, Model 5 (221 channels) achieves close agreement with prior theoretical and experimental data sets at intermediate angles (30°–120°), particularly at 30 eV. However, discrepancies persist at extreme forward and backward angles. Above 12 eV, Model 5 presents a smooth decay, aligning with the redistributive scattering from open inelastic channels.

Inelastic scattering reveals slightly distinct behaviors between the first (^3^ *A*′) and second (^1^ *A*′) excited states. Both excited states exhibit pronounced structures below 12 eV, a sharp decline of magnitude at 12 eV and smoother decay up to 30 eV. In contrast, the ^1^ *A*′ state shows greater angular isotropy in the DCS compared to the ^3^ *A*′ state DCS with Model 3 (183 channels) unexpectedly yielding lower cross sections than Model 5 at 20 and 30 eV. Despite these state-specific differences, a comparison of elastic and inelastic results robustly confirms channel count as the dominant factor in cross-section suppression, with models with similar channel counts yielding similar cross section magnitude and shape, particularly for the elastic scattering.

Despite THF’s central role as a DNA sugar analog, no experimental or theoretical data exist for its individual electronically inelastic scattering channels. Our systematic characterization of the first two lowest excited states therefore provides the first and only available benchmarks for low-energy electron interactions in this biologically relevant system. While elastic scattering results align with prior data sets, the absence of inelastic comparisons necessitates reliance on internal model consistency, where exhaustive channel coupling (Model 5) emerges as the most rigorous approach. These findings establish a foundation for understanding electron-driven damage mechanisms in complex biomolecules. Future work should expand channel counts to resolve residual anomalies and incorporate condensed-phase effects, such as solvation and dissipation, to bridge the gap between gas-phase calculations and realistic cellular environments.
